# Black US women share their experiences with follow-up after abnormal cervical cancer screening

**DOI:** 10.1016/j.puhip.2025.100658

**Published:** 2025-10-01

**Authors:** Ariel Washington, Kassidy F. Houck, Bridget W. Nelson, Diane M. Harper

**Affiliations:** a Department of Oncology, Population Science, Wayne State University, Detroit, MI, USA; b Department of Epidemiology, School of Public Health, University of Michigan, Ann Arbor, MI, USA; c Departments of Family Medicine, Obstetrics&Gynecology and Women's and Gender Studies, University of Michigan, USA

## Abstract

**Objectives:**

Black women have both high screening and high cancer rates, indicating a lack of appropriate follow-up after an abnormal screening. Our mixed methods study explores the experiences of Black women who had abnormal cervical cancer screening results in Michigan.

**Study design:**

Sequential mixed-method study design.

**Methods:**

We identified a random group of 72 Black women with recent abnormal screening results requiring colposcopy follow-up from the aggregated local healthcare systems and invited them to participate. We designed a quantitative survey based on validated national health survey modules and a qualitative interview structured on the Theoretical Domains Framework (TDF), which was analyzed using Applied Thematic Analysis (ATA). We calculated each person's social deprivation index (SDI) based on quantitative results.

**Results:**

Fourteen women completed the survey and interview. Five, seven, and two women chose no colposcopy, appropriate colposcopy, and missed colposcopy, but returned for close interval surveillance, respectively. The qualitative themes provided a potent emerging theme: the eternal cycle of human papillomavirus (HPV) uncertainty, which provided context for the other identified themes centered around lack of knowledge leading to emotional burdens, which intersected with being seen as a Black woman. We found similar barriers that have been noted for screening, such as relationships, positive and negative, having some influence on follow-up behavior.

**Conclusions:**

With the shift to primary HPV screening, new educational platforms must be created and tailored to explain the HPV cycle for each racial/ethnic community.

**Precis:**

The lack of knowledge about the eternal cycle of HPV uncertainty leads to Black women's lack of colposcopy follow-up.

## 1 Introduction

Healthy People 2030 has set a goal of 84.3 % of women 21–65 years old to be up-to-date with a cervical cancer screen [1]. The 2018 Behavioral Risk Factor Surveillance System (BRFSS) results show that Black women exceeded the goal at 87.1 %, the highest of all races [2,3]^.^ Yet cancer rates continued to be higher for Black women at 16.8/100,000 compared to Whites at 10/100,000 [4].

Abnormal cervical cancer screens require a diagnostic workup. The two US follow-up options include colposcopy or repeat surveillance in one year. Referral to colposcopy means that the immediate risk of cervical intraepithelial neoplasia grade 3 or worse (CIN3+) is at or greater than the 4 % threshold set by the 2019 management guidelines [5].

The New Mexico HPV Pap Registry (NMHPR) showed that the percentage of all women lacking a guideline-recommended follow-up colposcopic biopsy depended on the actual screening result, not the risk-based threshold, and ranged from 31 % for positive HPV 16/18 results to 90 % for high-grade (HSIL) cytology results [6]. Meta-analyses on follow-up studies reveal a substantial lack of follow-up in all cervical cancer screening programs globally, regardless of the technique for screening, and without racial intersectionality analyses [7].

In survey studies, Black women, compared to White women, have less recall about being informed of abnormal results or being called back for colposcopy [8,9]. As cervical cancer screening shifts to primary HPV screening without cytology, we are concerned that Black women may be further disadvantaged in appropriate care after a positive HPV test. Our mixed methods study explores the experiences of women who self-describe as Black, who made decisions to follow up or not after a cervical cancer screening result that would lead to a colposcopy recommendation.

## 2 Methods

This sequential mixed method study design started with a quantitative data/chart review leading to a qualitative interview invitation, where the qualitative interview is grounded in Applied Thematic Analysis (ATA), a systematic approach to analyzing qualitative data and theme development that delves into the perceived barriers and facilitators of follow-up screening [10]^.^ The University of Michigan Institutional Review Board (HUM00242983) approved the study from July 1, 2024, through October 31, 2024. The study followed the Standards for Reporting Qualitative Research (SRQR) standards for reporting [**SRQR Checklist supplement**].

## 3 Population

People with a cervix between 21 and 65 years old with a self-identified race containing Black or African American in the health record who received cervical cancer screening in a Midwest health system between January 1, 2021, and December 31, 2023, were eligible for study review. If their cervical cancer screen were abnormal, leading to a risk of immediate CIN 3+ disease at 4 % or greater, women would have been clinically referred to colposcopy and qualified for recruitment to our study. The risk of immediate CIN3+ was determined by the 2019 ASCCP Clinical Management Guidelines for age groups 30–65 and 21–29, followed by the health system [11] (Supplemental Tables 1 and 2). We randomly selected 72 Black women whose medical record review indicated cervical cancer screening and a result requiring a colposcopy; 14 (19 %) accepted the interview.

Exclusion criteria included being pregnant at the time of the screening, no further health system visits after the screening exam, screening intended for vaginal or vulvar disease, a diagnosis of cervical cancer after screening, and a hysterectomy or surgical excision of the cervix (trachelectomy).

We defined three response groups. The first two were those with and without appropriate colposcopy follow-up. If a colposcopy occurred at any time before the next screening, the group was considered appropriately followed up, regardless of the time between screening and colposcopy. The third group of women did not follow up on their recommended colposcopy but did return for further surveillance at an interval earlier than routine screening (Table 1).

**Table 1:** Screening results and decisions among interviewees.

No Follow-up								
	Index Screen		Decision	Surveillance		Decision	Surveillance	
13	HPV 16		NILM	No Colpo		None		
33	HPV 12other		ASC-H	No Colpo		HPV 12other	ASC-H	
21	∗∗HPV12other		ASCUS	No Colpo		None		
19	HPV 12other	ASCUS	No Colpo	HPV 12other	LSIL	No Colpo	HPV negative	NILM
2	HPV 12other	ASCUS	No Colpo	None				
NILM means negative for intraepithelial lesion or malignancy. ASCUS means atypical squamous cells of undetermined significance ASC-H means atypical squamous cells cannot rule out high-grade disease LSIL means low-grade squamous intraepithelial lesion Surveillance occurred at the one-year mark. ∗∗means second HPV12other repeat screen								

Appropriate Follow-up								
	Index Screen		Decision	Bx Result	Surveillance		Decision	Bx Result
11	HPV 18	ASCUS	Colpo	Normal	HPV 18	LSIL	Colpo	Normal
48	∗∗∗HPV12other	NILM	Colpo	Normal	None			
61	∗∗HPV12other	NILM	Colpo	Normal	None			
72	∗∗HPV12other	NILM	Colpo	Normal	HPV negative	NILM		
60	∗∗HPV12other	NILM	Colpo	Normal	HPV negative	NILM	surveil	
56	HPV 12other	LSIL	Colpo	CIN2	HPV12 other	NILM	LEEP	CIN2
71	<30 yrs	ASC-H	Colpo	CIN2	HPV negative	NILM	surveil	
NILM means negative for intraepithelial lesion or malignancy. ASCUS means atypical squamous cells of undetermined significance ASC-H means atypical squamous cells cannot rule out high-grade disease LSIL means low-grade squamous intraepithelial lesion Surveillance occurred at the one-year mark. Colpo means colposcopy LEEP means loop electrosurgical excision procedure CIN2 means cervical intraepithelial neoplasia grade 2 ∗∗means second HPV12other repeat screen ∗∗∗means third HPV12other repeat screen								

Missed colposcopy but did come for further surveillance							
	Index Screen		Decision	Surveillance		Decision/Surveillance	
38	HPV 12 other	LSIL	No colpo	HPV negative	ASCUS	HPV negative	NILM
45	HPV 12other	LSIL	No colpo	HPV 12other	LSIL	Colpo	Normal bx
NILM means negative for intraepithelial lesion or malignancy. ASCUS means atypical squamous cells of undetermined significance LSIL means low-grade squamous intraepithelial lesion Bx means the biopsy taken during colposcopy							

## 4 Recruitment and data collection

Participants were recruited from the medical charts of an academic medical center in the Midwest United States. Each participant who agreed to be interviewed signed an electronic consent form. She could opt out of the audio or video recordings, but had to allow the interviewer to take notes of the conversation at a minimum. Interviews were conducted by two research assistants (KFH, BWN), who were trained by (AW) in qualitative interviewing, memoing, and analysis. The female study team (KFH, BWN) knew no participants before the study. All interviewees received a $50 incentive. The study team interviewed fourteen unique participants once between July 2024 and October 2024 in a one-on-one audio or video format, ensuring privacy. Three of the six women who preferred phone interviews did not consent to recording; instead, they required note-taking and memoing [12]. Eight of the eleven women who consented to recording preferred the Zoom interview. The interviews lasted an average of 26 min (16–52 min) [Supplemental Figure 1]. The TDF informed the semi-structured interview guide, covering thirteen domains pertinent to influencing behavior [Supplemental Figure 2].

### 4.1 Quantitative survey

All participants completed our quantitative survey based on the Health Information National Trends Survey (HINTS) [13], which regularly collects nationally representative data about the American public's knowledge of, attitudes toward, and use of cancer- and health-related information. We used demographics, cervical cancer, and health communication modules. The Social Deprivation Index (SDI) incorporates seven demographic characteristics from the American Community Survey into a single validated measure of a Social Determinant of Health where the highest numbers are the most deprived. We transformed the zip codes into aggregated zip code tabulation areas (ZCTAs) and SDI scores [14]. We used descriptive statistics of central tendency measures, frequencies, and distributions, as well as Chi-square and Fisher's exact tests to describe our population, using Statistica software, version 14.0 [15].

### 4.2 Qualitative analysis

Recorded interviews were transcribed verbatim, with detailed notes of all interview sessions. The authors met periodically to debrief about the interviews and analysis process and discuss the nascent theme and subthemes' development. Data was coded line by line and then transferred to Excel to develop codes inductively through consensus discussion and deductively using the TDF domain questions.

### 4.3 Authors' positionality

The research team for this study consisted of an experienced clinician-scientist (DMH), an experienced mixed methods researcher (AW), and two research assistants (KH and BN). AW, who identifies as a Black woman, supervised the data collection and the qualitative analysis. Interviews were conducted by KH and BN, both of whom identify as women and were trained on qualitative interviewing techniques.

## 5 Results

### 5.1 Population

Of the 14 interviewees from the 72 randomly selected Black eligible women, 5/30 had "no colposcopy follow-up," 7/31 had "colposcopy follow-up," and 2/11 had "missed colposcopy follow-up but had close interval surveillance." Their qualifying screening results are presented in Table 1. No difference existed between the random population meeting the inclusion/exclusion criteria and the interviewed population (Supplemental Table 3).

### 5.2 Quantitative survey

**Demographics**. All but one participant was divorced, widowed, separated, or never married across all three follow-up categories. Most were employed, two reported being disabled, and others were retired, students, or unemployed (Table 2).

**Table 2:** Characteristics and beliefs of interviewees.

	Total population	No Follow-up	Appropriate Follow-up	Missed colposcopy/came for surveillance
DEMOGRAPHICS	N = 14	N = 5	N = 7	N = 2
**Age, yrs (mean, SD)**	40.0 (10.4)	45.2 (12.5)	35.7 (8.8)	42.0 (5.7)
**Marital Status**				
Divorced	3 (21.4)	1 (20.0)	1 (14.3)	1 (50.0)
Widowed	1 (7.1)	0	1 (14.3)	0
Never been married	8 (57.1)	3 (60.0)	4 (57.1)	1 (50.0)
Separated	1 (7.1)	1 (20.0)	0	0
Living with a partner	1 (7.1)	0	1 (14.3)	0
**How many in the household, including yourself**				
1	4 (28.6)	2 (40.0)	1 (14.3)	1 (50.0)
2	6 (42.9)	1 (20.0)	4 (57.1)	1 (50.0)
3	3 (21.4)	1 (20.0)	2 (28.6)	0
5	1 (7.1)	1 (20.0)	0	0
**Children under 18 years in the household**				
0	7 (50.0)	3 (60.0)	3 (42.9)	1 (50.0)
1	5 (35.7)	0	4 (57.1)	1 (50.0)
2	2 (14.3)	2 (40.0)	0	0
**Education**				
High school graduate	1 (7.1)	1 (20.0)	0	0
Some college	4 (28.6)	2 (40.0)	2 (28.6)	0
College graduate or more	8 (57.1)	2 (40.0)	4 (57.1)	2 (100.0)
**Occupation**				
Employed	9 (64.3)	2 (40.0)	5 (71.4)	2 (100.0)
Unemployed	1 (7.1)	1 (20.0)	0	0
Student	1 (7.1)	0	1 (14.3)	0
Retired	1 (7.1)	1 (20.0)	0	0
Disabled	2 (14.3)	1 (20.0)	1 (14.3)	0
**Housing**				
Own	5 (35.7)	2 (40.0)	3 (42.9)	0
Rent	7 (50.0)	3 (60.0)	2 (28.6)	2 (100.0)
Occupied without paying monetary rent	2 (14.3)	0	2 (28.6)	0
**Social Deprivation Index**^a^				
15	1 (7.1)	0	0	1 (50.0)
20	1 (7.1)	1 (20.0)	0	0
45	3 (21.4)	2 (40.0)	0	1 (50.0)
*52*	1 (7.1)	0	1 (14.3)	0
*63*	1 (7.1)	0	1 (14.3)	0
*64*	2 (14.3)	0	2 (28.6)	0
*70*	2 (14.3)	0	2 (28.6)	0
*74*	1 (7.1)	1 (20.0)	0	0
*75*	1 (7.1)	0	1 (14.3)	0
*97*	1 (7.1)	1 (20.0)	0	0

**CERVICAL CANCER SCREENING**				

**Have you ever heard of human papillomavirus, HPV, not HIV, HSV, or herpes?**				
Yes	13 (92.9)	4 (80.0)	7 (100.0)	2 (100.0)
No	1 (7.1)	1 (20.0)	0	0
**Where have you heard about HPV?**				
Internet	7	1	6	0
School	2	1	1	0
Don't remember	1	1	0	0
Other	8	1	5	2
**Has a healthcare provider, such as a doctor or nurse, ever talked to you about an HPV test?**				
Yes	10 (71.4)	4 (80.0)	5 (71.4)	1 (50.0)
No	4 (28.6)	1 (20.0)	2 (28.6)	1 (50.0)
**Have you been told by your healthcare provider that you had an HPV infection?**				
Yes	11 (84.6)	3 (60.0)	6 (85.7)	2 (100.0)
No	2 (15.4)	2 (40.0)	0	0
**What happens after an HPV infection?**				
Requires medical treatment	5 (38.5)	2 (50.0)	2 (28.6)	1 (50.0)
Usually, go away on its own	8 (61.5)	2 (50.0)	5 (71.4)	1 (50.0)
**Do you think HPV can cause cervical cancer?**				
Yes	13 (92.9)	4 (80.0)	7 (100.0)	2 (100.0)
No	1 (7.1)	1 (20.0)	0	0
**What is the main reason you had your most recent Pap test?**				
An annual routine physical	13 (92.9)	5 (100.0)	6 (85.7)	2 (100.0)
Last Pap test was not normal	1 (7.1)	0	1 (14.3)	0
**How worried are you about getting cancer?**				
Not at all	1 (7.1)	1 (20.0)	0	0
Slightly	5 (35.7)	2 (40.0)	2 (28.6)	1 (50.0)
Somewhat	2 (14.3)	0	2 (28.6)	0
Moderately	1 (7.1)	0	1 (14.3)	0
Extremely	5 (35.7)	2 (40.0)	2 (28.6)	1 (50.0)

**HEALTHCARE COMMUNICATION**				

**How often did they explain things about your cervical cancer screening in a way you could understand?**				
Always	7 (50.0)	3 (60.0)	2 (28.6)	2 (100.0)
Usually	4 (28.6)	1 (20.0)	3 (42.9)	0
Sometimes	3 (21.4)	1 (20.0)	2 (28.6)	0
**How often did they involve you in decisions about what to do with the results of your cervical cancer screening as much as you wanted?**				
Always	8 (66.7)	2 (50.0)	4 (57.1)	2 (100.0)
Usually	2 (16.7)	1 (25.0)	1 (14.3)	0
Sometimes	1 (8.3)	0	1 (14.3)	0
Never	1 (8.3)	1 (25.0)	0	0
**How often did they make sure you understood your cervical cancer screening for you to take care of your health?**				
Always	10 (75.9)	3 (75.0)	5 (71.4)	2 (100.0)
Usually	1 (7.7)	0	1 (14.3)	0
Sometimes	1 (7.7)	0	1 (14.3)	0
Never	1 (7.7)	1 (25.0)	0	0

The higher the number, the more deprived, the poorer, the worse the social setting (n = 12). The lower the number, the less deprived, the richer, the better the social setting (n = 2).

a Social deprivation index (SDI). Robert Graham Center - Policy Studies in Family Medicine & Primary Care. (2018, November 5). Retrieved November 29, 2021, from https://www.graham-center.org/rgc/maps-data-tools/sdi/social-deprivation-index.html.

There was a robust distribution of SDI from the least to the most socially deprived. All participants in the "appropriate follow-up" group were from the higher SDI regions with the most social deprivation. The "no-follow-up" group was split into two with the highest and three with the lowest SDI scores. The "missed follow-up" group had two participants with the lowest deprivation scores.

### 5.3 Knowledge – HPV and cervical cancer

Almost all respondents in our study indicated they had heard of HPV in the past from a variety of sources. The internet was the most common source for participants who did follow up. However, 28.6 % of the respondents indicated that their healthcare provider **never** mentioned an HPV test, and 40 % of those not following up thought they had **never** been told they had an HPV infection. The natural history of HPV was interpreted differently by all three follow-up groups. Half of those with "no follow-up," 71.4 % of those with "appropriate follow-up," and half of those who "missed their colposcopy but returned for surveillance" misunderstood the natural clearance of HPV. Nearly all correctly believed that HPV could cause cervical cancer. All who repeatedly had abnormal screens incorrectly believed the main reason for their most recent Pap test was not because their prior screen was abnormal, but for routine annual screening.

Respondents with "no follow-up" expressed the extremes of worry about getting cancer. All respondents with "appropriate follow-up" and "missed colposcopy but continued surveillance" were more worried about getting cancer than not (p < 0.05).

**Health communication**. The misunderstandings around personal risk came from either the inaccurate physicians' explanations or misinterpretation of what the physician said, as respondents reported that their healthcare providers explained the cervical cancer screening process to them most of the time (92.3 %) and involved them in decision-making as much as they wanted about the needed follow-up (91.7 %). Yet, still, they missed the required colposcopy.

Nonetheless, the participants who received the correct information from their physicians but had "no follow-up" responded that some healthcare providers always (75 %) made sure they understood their cervical cancer screening results and next steps, yet they did not follow up.

### 5.4 Qualitative interviews

The participants' interviews reinforced the quantitative survey results, providing more insightful themes of 1) The eternal cycle of HPV uncertainty. 2) Lack of Understanding whose subthemes are: lack of accurate information, lack of understanding of the natural history of persistent HPV, lack of understanding of the test results, and lack of understanding of subsequent actions. 3) Relationships, positive and negative, with subthemes of patient with the provider, healthcare system (access, cost), social support, and self. 4) The emotional burden of an abnormal screening test with subthemes of distress, anxiety, sadness magnified by being Black, and shame. 5) The influence of Black identity on the experience (Table 3).

**Table 3:** Themes from the qualitative interviews.

Theme (subtheme)	Decision	Quote
**Eternal cycle of HPV uncertainty**	**Appropriate follow-up**	*…****is it necessary?****How long has it been bothering you? How much is it going to cost? Because everything is deductible-based these days … we gotta be like negotiators when we have these appointments … It depends on the deductible (ID48)*
	**No follow-up**	*… I told you years ago I had abnormal paps … And it was nothing back then … See you in a couple of months …. (ID 33)*
		*… I really didn't make any decisions based off an abnormal result, because I feel like every Pap smear that I've ever had … came back abnormal, so [it did not need my] urgent attention … while they said it's abnormal, .. at this point it's pretty normal for me. (ID 21)*
		*… When I first had one, many many years ago, I felt scared, but as the years went on, it became less frightening. (ID 2)*
**Lack of Understanding**		
**a. Physician's lack of correct knowledge**	**Missed colposcopy**	*. I was told****it was not concerning cancer strains****, and****since I didn't have the strains I wouldn't look for information****(ID38)*
		*..I just make sure I am doing the****yearly Pap****… the whole idea of having a colposcopy is uncomfortable … (ID45)*
	**No follow-up**	*.., but the doctor .. stated that I had a 6 % chance of developing cervical cancer in the next five years and recommended follow-up in a year.(ID19)*
		*When they say abnormal, I'm thinking, is something wrong? Because … it's always just like, yeah,****follow up in a couple of months****, and we'll see how you're doing.****There's never really an explanation of what that really means.****(ID 33)*
		*I have Lupus, and the Lupus doctor said I was due. then the Lupus nurses called and said****the results can mean different things;****it doesn't always mean bad, sometimes it can be they didn't get an accurate result, and you need to get it redone … no mention of precancer/cancer (ID 13)*
**b. Patient's lack of understanding of the natural history of persistent HPV infections**	**Missed colposcopy**	*I was worried [after] my first one ..kind of surprised that [next cervical cancer screening test results] everything looked good. (ID45)*
	**No follow-up**	*… After initially having abnormals 20 years ago I didn't have anymore until about 7-8 years ago****and was just confused how I got it again and kept testing positive for HPV.****(ID19)*
**c. Lack of understanding of the test and its interpretation**	**Appropriate follow-up**	*There has been so much advertisement about HPV, but I****realized I hadn't received a lot of education****. And so once I realized that I had it [HPV], I felt confused, afraid … (ID56)*
		*… I like for them****to call me****.. Sometimes it's too much. I****hate to read stuff****. (ID 72)*
		*… I kind of wish they would explain a little bit more, you know, like exactly what is going on, if I get this type of result. (ID 61)*
	**No follow-up**	*…****But I'm not sure if that's because they're screening for cervical cancer or if it was just a part of the Pap smear.****(ID 2)*
		*. You know,****I'm following that because of my, you know, because of my own feelings …****But****I do wish, like, I want them to kind of explain things more to me****. (ID 61)*
**Relationships**		
**a. Patient with Provider**	**Appropriate follow-up**	*… I was very well prepared. Like very like, when she took the second Pap smear, we had already talked about like worst case scenario. And so, you know, when it came back, I wasn't. It wasn't scary. It was like, okay, you know. I know what's next. (ID48)*
	**Missed colposcopy**	*… Knowing that I had my primary care provider and doctors that supported me and my decisions and never made me feel forced to do anything and having discussions and understanding where I come from..(ID38)*
**b. Patient with Health Care System**		
**i. Access**	**Appropriate follow-up**	*And I think because I've had more positive experiences, it's made me;****I'm more likely to follow up and get procedures done that I know that I need to get done, especially vaginal things, which are my absolute least favorite, to ensure, you know, that I'm getting care****. (ID 56)*
		*It's always been easy to get my Pap smears, and it was very easy to get the colposcopy. (ID 48)*
		*But yeah, I had honestly forgot about that [needing a colposcopy] ..****nobody had called me to schedule that, even though my doctor had told me that they would be following up.****(ID11)*
	**No follow-up**	*… when the colposcopy people called … the lupus infusion schedule people [changed infusions] now to weekly, so that made scheduling and keeping appointments difficult (ID13).*

**ii. Cost**	**Missed colposcopy**	*.. for something like that I would definitely try my best to get in when something is wrong. Our health is more important, we need the jobs but****health has to trump our jobs****. We get replaced in a heartbeat if we can not work so we have to take care of our health first. (ID 38)*
		*… I am a nurse so I have good health literacy to follow up, just knowing that I have****access with insurance****is reassuring. (ID45)*
	**No follow-up**	*… I had lost my health insurance, I had medicaid before, but my income is now too high. So I lost my insurance for a couple of months, and the insurance for my job was unaffordable for me, so I did not have insurance for a period of time. I ended up being able to get it through my second job,****so my procedure was delayed because I didn't have insurance****. My insurance that I have now is through my primary job. The second job enables me to pay for my insurance through my main job****. If I hadn't gotten insurance I wouldn't have gone because I lost my medicaid earlier this year in January and cannot afford the screening otherwise****. (ID19)*
**c. Patient with Social Support**	**Appropriate follow-up**	*… My friends let me feel more comfortable. You know she's like, 'oh, it's not that bad. I promise you I had the same thing done.' And I'm like, oh, okay. So she kind of you know,****she made me feel more comfortable****. (ID 61)*
		*… But yeah, it, you know. Honestly, it****was through my friend kind of giving me a duh momen****t. This is why we get these. [colposcopy]. things done. (ID56)*
		*… My sister.. we were having a conversation at her house.. we both have HPV … she said 'Continue to check it. We don't want to get worse. We might have to be treated now'. (ID60)*
**d. Patient with Self Empowerment**	**Appropriate follow-up**	*… I believe in keeping up with my health as much as possible. (ID 2)*
		*… I always go to the doctor. I'm just like, when I get any type of pain or anything. I'm at the doctor, because you never know. That's just me myself. People like me live at the doctor.****I'm gonna go****. Anything new that's different.(ID 11)*
		*… it was a pretty easy decision to come to, just because I care about my own health. And****I've had a different form of cancer when I was younger.****So I just want to make sure I don't get cancer again in any shape or form.(ID71)*
**Emotional Burden**		
**a. Distressing**	**Appropriate follow-up**	*…. I was very nervous, but I'm a suppressor. So it's kind of like, okay, you gotta go … you work to make..the best of the situation. (ID48)*
	**No follow-up**	*… Honestly, I hate getting test results, like, it just feels like it's just another weight piled onto my shoulder … (ID 21)*
**b. Anxious**	**Appropriate follow-up**	*… I was genuinely scared that I absolutely have cancer … (ID60)*
		*… Just trusting that if I just follow what I'm supposed to follow, that you know everything will work out on its own. (ID71)*
	**No follow-up**	*… I am nervous about getting the follow-up done and [keep] pushing it back to a later date. (ID 13)*
**c. Sadness**		
**i. Black Identity**	**Appropriate follow-up**	*I think I'm very on guard with invasive procedures, because I don't know if the racial narratives, which could be implicit or not, could impact the way that I am treated, less so in communication, but more so in terms of more invasive procedures, which includes pap smears. I tend to be like, uncomfortable in those, because they're generally uncomfortable, but I'm also at times nervous about how the physician is engaging my body, and how they perceive me, and what they think my threshold for pain, or again, invasiveness may be. (ID 56)* *But I will say that being Black, I always have to ensure that I am my own best advocate for my health, because I know that oftentimes Black people, and Black women especially can be told to you know. Wait and see what happens if they, you know, have symptoms, or you know, maybe given the brush off with certain things, or told that you know certain symptoms that they have may be related to stress or diet, or whatever the case may be, when, in fact, it could actually be something much bigger. (ID 11)*
	**Missed colposcopy**	*… I was devastated, it stressed me out and made me very sad. It makes you feel dirty or have feelings of "who will want me now?" …. Alot of shame and sadness. It's depressing, no one wants to get that news. It's a thing that makes you feel isolated and people will shun you if they know.. (ID 38)*
**d. Shame**	**Appropriate follow-up**	*… you know, like feeling ruined … Like, have I … just****like impacted my sexual health in a way that is irreversible?****(ID56)*
		*… But at first it was just a lot of a lot of fear, a lot of anxiety, a lot of self-blame … (ID56)*
**Black Identity**		
	**Appropriate follow-up**	*… I think I'm****very on guard with invasive procedures because I don't know if the racial narratives, which could be implicit or not, could impact the way that I am treated, less so in communication but more so in terms of more invasive procedures, which include pap smears****…. (ID 56)*
		*… But I will say that being Black, I always have to ensure that I am my own best advocate for my health because I know …****Black women especially, can be told to you know. "Wait and see what happens." you may be given the brush off with certain things****… when, in fact, it could actually be something much bigger. (ID 11)*
	**Missed colposcopy**	*I don't feel like I, as a Black woman, am being heard. I feel like my concerns are just brushed off. A little frustrating with that, concerns not being fully investigated and looked into. (ID45)*
	**No follow-up**	*Black people need to be included [in research] because I also had an experience [where] it doesn't work for Black people. (ID 2)*

**Eternal Cycle of HPV Uncertainty**. Oftentimes, women reported having had multiple positive HPV tests interspersed with negative HPV tests in the past. Guidelines for the management of abnormal cervical cancer screening have created this eternal cycle of HPV uncertainty that has no definite step to help women be wholly cured of HPV. Therefore, many women will be in a yearly repeat surveillance cycle because of persistent HPV infection for much of their lives.

Repeated HPV infections may lead to anxiety (next theme) but also compliance with colposcopy. Or participants indicated that being HPV positive was old news for which they did not feel any urgent need for action and became less concerned (rather than more) with the more HPV-positive results they had. Participants stuck in this cycle described their sense of urgency decreasing over time, as they no longer perceived an abnormal result as an urgent concern. In this study, women discussed how they had had an abnormal result before and did not think it warranted a follow-up or was a serious concern. For those women in particular, being stuck in the eternal cycle devalued the seriousness of an abnormal result and discouraged the women from following up.

**Lack of understanding**. Participants made multiple comments pointing to a lack of accurate information from the physician to the patient, with concomitant misunderstandings by the patient regarding both the disease and the colposcopy follow-up steps, all resulting in missed or no colposcopy.

Multiple participants mentioned that they did not understand the test result or what to do about it, resulting in some following -up and others not. Many spoke as if the Pap smear was different from the cervical cancer screening.

**Lack of clarity** around the severity of the abnormal result and lack of awareness and knowledge about the abnormal result were additional roadblocks to scheduling for follow-up. Several women were unaware and unsure of how serious an abnormal result was and were uncertain if they were even supposed to follow up after receiving the result. A clear lack of education about the topic made it difficult for some women to gauge the seriousness of the abnormal result.

**Relationships are influential to behavior**. Relationships, both positive and negative, influenced behavior. The patient-provider relationship was a crucial factor that impacted willingness to follow-up after an abnormal result. Aspects of the relationship that negatively impacted willingness to follow-up included the level of communication between patient and provider, the medical office and healthcare system, and the level of support felt by the patient from the provider. When the patient-physician relationship was positive, women were open to colposcopy and returned for follow-up. Healthcare providers who were encouraging and willing to educate their patients on the importance of following up and making the next step of the process easy were important in influencing women to schedule and attend additional appointments.

The patient-healthcare system relationship was defined by access and cost. Positive experiences with setting appointments led to appropriate follow-up, whereas scheduling appointments for different medical needs or lack of return calls from scheduling led to no follow-up. Importantly, the financial cost of colposcopy was critical for those who missed or had no follow-up.

**Emotional well-being and distress**. Nearly all participants shared an emotional burden felt from having an abnormal cervical cancer screen. Across all three follow-up options, participants discussed the relationship between screening and their emotional well-being. The emotional distress, worry, and anxiety produced in response to the concern of a possible cancer confirmation impacted their willingness to follow up. This distress, worry, and anxiety acted as an emotional roadblock to scheduling and attending a colposcopy appointment. Others used the emotional responses as motivation for follow-up. As the burden progressed to anxiety, women did not miss surveillance later.

As anxiety turned to sadness, there was a component of Black identity intermixed. Black women felt judged about their sexual activity. Other times, Black women felt socially isolated because of their abnormal cervical cancer screening and missed their colposcopy. Shame leads to new perceptions of sexual harm from their completed colposcopy.

**Role of Social Identity and Perception in Decision Making and Healthcare Engagement**. Social identity and navigating the healthcare system as Black women was a complicated topic for the participants. There was a wide variety of experiences for those who participated in this study, from some who have said they have never experienced overt forms of racism and mistreatment within the healthcare system to those who have felt like test subjects within their encounters. Because of the past collective history of Black people and Black women with the medical system, several of the women felt as if they needed to be on guard and protective of their bodies. This awareness of the impact of Black identity while navigating the healthcare system caused some participants to be deliberate in gathering information and completing follow-ups. Additionally, because they were Black, their concerns were brushed off, unanswered, or they were devalued, leading to missed colposcopies.

## 6 Discussion

Black women, in particular, skip colposcopy [16]^.^ Our work is the first to detail the quantitative and qualitative study of all three follow-up behaviors among Black women after an abnormal cervical cancer screen requiring colposcopy.

A new, unique qualitative theme consistently appeared: "The Eternal Cycle of HPV Uncertainty." In prior mixed methods work, no set of women have expressed ongoing concern about an infectious disease that has no cure but may cause cancer with waffling (positive/negative/positive) results test after test.

Black women tire of repeated tests and invasive exams, especially when the Black experience has included inappropriate, repeated, invasive medical procedures for other ailments. They gain no further information about their status and develop the ennui of a positive HPV test. Biomarkers and advanced genomics are needed to break this cycle into acceptable follow-up intervals. HPV infection cures and HPV pre-cancer cures are desperately needed.

The change to primary HPV testing away from "Pap" is a testament to science over the past 20 years [17]. However, as it has allowed changes in the screening technique, interval, commencement, and cessation ages for screening, it has also created confusion. Continuous scientific updates confused the electronic healthcare logistics for physicians by repeatedly changing ordering and follow-up routines [18].

With confusion comes a lack of understanding for both the physician and the patient, leading to some level of misunderstanding about some aspect of the process, as noted for other cancer screening tests [19]. The physician did not offer the correct recommendation or did not interpret the test result correctly, or the patient did not understand HPV as a persistent infection with a natural history of increasing likelihood of pre-cancerous transformation as the number of positive tests increased over time.

The participants' discordant quantitative knowledge scores about HPV, HPV infection, an HPV test, and cervical cancer are quite concerning in our population. They demand a more tailored and comprehensive cervical cancer education program for communities of Black women.

Sometimes, knowledge and understanding undermined the women's decisional process. Unlike the decision to screen, which is highly correlated to social deprivation indices [20,21], we showed that the SDI, a cumulative measure of many barriers, was unrelated to decisional action. Some highly educated women with a low SDI took a calculated risk of no colposcopy but self-created a safety net by a surveillance return. On the other hand, some women with low education and high SDI did have their colposcopy but without any understanding of what was happening.

Anxiety, worry, shame, and emotional distress will always play a role in any positive cancer screening test [7,16,22,23]. Still, for cervical cancer prevention in our Black interviewees, such distress seemed secondary to the anxiety of not understanding what was going on. Appropriate education tailored to the community, by respected community health educators, by the woman's trusted physician (provided the physician has the correct information), and reinforced by their social groups may increase the rate of colposcopy follow-up after specific positive HPV tests.

### 6.1 Strengths and limitations

A strength of this work is the identification of the eternal cycle of HPV uncertainty as a central theme for modifying follow-up behavior after an abnormal cervical cancer screen. The second strength of this work is the full spectrum of social deprivation from none to worst among the Black participants and the detailed access to their medical records for gynecologic care linked throughout the region.

A weakness of our study is the limited number of interviews we conducted for each follow-up group. However, we showed no demographic difference between the interviewees and non-interviewees, limiting non-participation bias. Additionally, the interview information is robust and saturated our resulting themes. These findings are not generalizable; however, they are transferable to similar settings and populations.[24]

## 7 Conclusions

The eternal cycle of HPV uncertainty must be addressed in tailored and authentic conversations to increase Black women's understanding of cervical cancer screening, giving access to correct information to drive their post-screening follow-up decisions.

## Consent to participate

Yes, women agreed to be interviewed.

## Financial disclosure

No financial disclosures were reported by the authors of this paper.

## Role of funding source

The funders did not influence the topic or data presented in this manuscript.

## Ethics approval

University of Michigan Institutional Review Board (HUM00242983), 1/31/2024.

## Consent to publication

Women agreed to have their unidentified comments published in aggregate after thematic analyses.

## Declaration of generative AI and AI-assisted technologies

None.

## Funding support

Blue Cross Blue Shield of Michigan Foundation, 23-PAF05454, UM1TR004404 (to Michigan Institute for Clinical and Health Research) and P30CA046592 (to the University of Michigan Rogel Cancer Center) from the National Institutes of health.

## Conflict of interest statement

No author has any conflict of interest to report.

## Abbreviations and Acronyms

(ASCCP): American Society of Colposcopy and Cervical Pathology
(ATA): Applied Thematic Analysis
(BRFSS): Behavioral Risk Factor Surveillance System
(CIN3+): Cervical Intraepithelial Neoplasia Grade 3 or worse
(HINTS): Health Information National Trends Survey
(HSIL): High-grade squamous intraepithelial lesion
(HPV): Human papillomavirus
(NMHPR): New Mexico HPV Pap Registry
(SDI): Social Deprivation Index
(SRQR): Standards for Reporting Qualitative Research
(TDF): Theoretical Domains Framework
(ZCTA): Zip Code Tabulation Area
