# Supplementary material for: Black US women share their experiences with follow-up after abnormal cervical cancer screening
Source: Public Health Pract (Oxf). 2025 Oct 1;10:100658. doi: 10.1016/j.puhip.2025.100658 (PMC12519130; doi:10.1016/j.puhip.2025.100658)
Supplement: Multimedia component 1 [file mmc1.docx]

***PHONE INTERVIEW***

*In this interview, we would like to find out what you thought about your past cervical cancer screening– how it was for you and what feedback you might have about improving the experience.*

*First, I’ll begin with some general questions about cervical cancer screening. Then, we’ll go through more details about your experience with screening. To finish, we’ll end with a few more questions about your overall healthcare experiences.*

*If there are any questions that you don’t want to answer, just say “skip,” and we’ll move on to the next one. And if there are any questions you don’t understand, please let me know, and I can ask it differently.*

GENERAL CERVICAL CANCER SCREENING

1. Please describe a previous pap smear experience. If your most recent experience is your only experience, please describe the reasons why. ?
   - **Probe**: Can you tell me how easy or difficult it was to schedule your appointment? Attend the screening?
2. Can you describe for me the reason(s) why you decided to undergo a pap smear test recently (or within the past 5 years)?
3. Can you describe the purpose of a pap smear test for me?

ABNORMAL CERVICAL CANCER SCREENING FOLLOW-UP

1. Can you describe what you believe an abnormal result means?
2. If you received abnormal results, can you describe for me the reasons why you did or did not return for additional testing?
   - What steps would you take after receiving an abnormal result?
3. How were your pap smear results communicated to you? What information was shared?
4. What next steps were communicated with you? Was there any support or help offered to you?
5. If/when you received an abnormal screening result, did you feel capable of seeking follow-up care?
6. Can you describe what did and did not make you feel capable of seeking follow-up care?
7. What do you believe will or would happen if you did seek follow-up care for an abnormal result?
8. What factors influence your decision about making or attending a medical appointment?
9. What factors influence your decision-making about making or attending a follow-up appointment for an abnormal result?
10. Did you search for any information about cervical cancer screening or follow-up information?
    - Where did you look for this information, and what did you learn from your search?
    - How did the information you read make you feel about follow-up testing?
    - If you didn’t search for any additional information, what are some of the reasons why?
11. Did you search for any information about the experiences of cervical cancer patients or survivors? What information did you find, and did you feel as if it was useful to you?
12. Thinking about other health scenarios, such as breast cancer screening or colonoscopy, what would prompt you to return for follow-up testing?
    - **Probes/Follow-up**: How are those same reasons different or similar to cervical cancer screening?
13. What strategies or support do you feel would be helpful from your provider or the health system that would make follow-up easier?
14. Do you view yourself as at risk for cervical cancer?

HEALTHCARE EXPERIENCE

The following questions are about your experiences navigating the healthcare system and your comfort with care.

1. How do you feel your social identity as a Black woman impacts your overall healthcare experience?
2. Thinking about a time when you felt comfortable with a healthcare visit, what stood out about that experience? How did you feel the provider and medical staff treated you?
3. Now, thinking about a time when you did not feel comfortable with a healthcare visit, what stood out about that experience? How did you feel the provider and medical staff treated you?
4. Is there anything else you would like to share with me? Any topic you think we didn’t cover?
