# Supplementary material for: Black US women share their experiences with follow-up after abnormal cervical cancer screening
Source: Public Health Pract (Oxf). 2025 Oct 1;10:100658. doi: 10.1016/j.puhip.2025.100658 (PMC12519130; doi:10.1016/j.puhip.2025.100658)
Supplement: Multimedia component 2 [file mmc2.docx]

**Theoretical Domains Framework (TDF) – Cervical Cancer Screening Questionnaire**

Target behavior: Follow-up after an abnormal cervical cancer screening test

Population: Black women in the general screening population

**Domains with Definition Domain Questions**

| **1. Knowledge** | **Interview Questions** |
| --- | --- |
| An awareness of the existence of something | - Can you describe for me the purpose of cervical cancer screening? - Can you describe what you believe an abnormal result means? |
| **2. Skills** |  |
| An ability or proficiency acquired through practice | - If you received abnormal results, can you describe to me the reasons why you did or did not return for additional testing?   - How did you feel coming to that decision? What thoughts or feelings do you think impacted this?   - What steps would you take after receiving an abnormal result? |
| **3. Social/professional role and identity** |  |
| A coherent set of behaviors and displayed personal qualities of an individual in a social or work setting. | - How do you feel your social identity, as a Black woman, impacts your overall healthcare experience? - What strategies or support would be helpful from your provider or the health system that would make follow-up easier? |
| **4. Beliefs about capabilities** |  |
| Acceptance of the truth, reality, or validity about an ability, talent, or facility that a person can put to constructive use | - Did you feel capable of seeking follow-up care if/when you received an abnormal screening result? - Can you describe what did and did not make you feel capable of seeking follow-up care? |
| **5. Optimism** |  |
| The confidence that things will happen for the better or that desired goals will be attained. |  |
| **6. Beliefs about consequences** |  |
| Acceptance of the truth, reality, or validity about outcomes of behavior in a given situation | - What do you believe will or would happen if you did seek follow-up care for an abnormal result? |
| **7. Reinforcement** |  |
| Increasing the probability of a response by arranging a dependent relationship, or contingency, between the response and a given stimulus | - What strategies or support would be helpful from your provider or the health system that would make follow-up easier? |
| **8. Intentions** |  |
| A conscious decision to perform a behavior or a resolve to act in a certain way | - Did you search for any information about cervical cancer screening or follow-up information?   - Where did you look for this information, and what did you learn from your search?   - How did the information you read make you feel about follow-up testing?   - If you didn’t search for any additional information, what are some of the reasons why? - Can you describe for me the reason(s) why you decided to undergo cervical cancer screening recently (or within the past 5 years)? |
| **9. Memory, attention, and decision processes** |  |
| The ability to retain information, focus selectively on aspects of the environment, and choose between two or more alternatives | - Please describe a previous cervical cancer screening experience. This may have involved a Pap smear and HPV test. If your most recent experience is your only experience, please describe the reasons why.   - **Probe**: Can you tell me how easy or difficult it was to schedule your appointment? Attend the screening? - What factors affect your decision about whether to make or attend a medical appointment? |
| **10. Environmental context and resources** |  |
| Any circumstance in a person’s situation or environment that discourages or encourages the development of skills and abilities, independence, social competence, and adaptive behavior | - What factors affect your decision about whether to make or attend a follow-up appointment for an abnormal result? |
| **11. Social influences** |  |
| Those interpersonal processes that can cause individuals to change their thoughts, feelings, or behaviors | - Thinking about a time when you felt comfortable with a healthcare visit, what stood out about that experience? How did you feel the provider and medical staff treated you? - Now, thinking about a time when you did not feel comfortable with a healthcare visit, what stood out about that experience? How did you feel the provider and medical staff treated you? |
| **12. Emotion** |  |
| A complex reaction pattern involving experiential, behavioral, and physiological elements by which the individual attempts to deal with a personally significant matter or event | - How were your pap smear results communicated to you? What information was shared?   - What next steps were communicated to you? Was there any support or help offered to you?   - What were your first or initial thoughts once receiving your result? How did you feel hearing the healthcare provider tell you that you had an abnormal result? |
| **13. Behavioral regulation** |  |
| Anything aimed at managing or changing objectively observed or measured actions | - How were your pap smear results communicated to you? What information was shared?   - What next steps were communicated to you? Was there any support or help offered to you?   - What were your first or initial thoughts once receiving your result? How did you feel hearing the healthcare provider tell you that you had an abnormal result? |
