# Supplementary material for: Black US women share their experiences with follow-up after abnormal cervical cancer screening
Source: Public Health Pract (Oxf). 2025 Oct 1;10:100658. doi: 10.1016/j.puhip.2025.100658 (PMC12519130; doi:10.1016/j.puhip.2025.100658)
Supplement: Multimedia component 3 [file mmc3.docx]

Supplemental Table 1. ASCCP 2021-2023 management for 30-65 year olds

| **Repeat HPV and cytology**  **in one year** | | **Repeat HPV and cytology**  **in 3 years** | | **Repeat HPV and cytology**  **in 5 years** | | **Colposcopy with Biopsy and Endocerivcal Curettage** | |
| --- | --- | --- | --- | --- | --- | --- | --- |
| HPV result | cytology result | HPV result | Cytology result | HPV result | Cytology result | HPV result | Cytology result |
| positive | NILM | negative | LSIL | negative | NILM | unknown/none | AGC |
| positive | NILM no TZ |  |  | negative | ASCUS | positive | AGC |
| positive 12 other | NILM |  |  | negative | NILM no TZ | negative | AGC |
| positive 12 other | NILM no TZ |  |  |  |  | unknown/none | ASC-H |
|  |  |  |  |  |  | negative | ASC-H |
|  |  |  |  |  |  | positive | ASC-H |
|  |  |  |  |  |  | positive | ASCUS |
|  |  |  |  |  |  | positive 16/18 | ASCUS |
|  |  |  |  |  |  | positive other 12 | ASCUS |
|  |  |  |  |  |  | unknown/none | HSIL |
|  |  |  |  |  |  | negative | HSIL |
|  |  |  |  |  |  | positive | HSIL |
|  |  |  |  |  |  | unknown/none | LSIL |
|  |  |  |  |  |  | positive | LSIL |
|  |  |  |  |  |  | positive 16/18 | LSIL |
|  |  |  |  |  |  | positive 12 other | LSIL |
|  |  |  |  |  |  | positive 16/18 | NILM |
|  |  |  |  |  |  | positive 16/18 | NILM no TZ |
|  |  |  |  |  |  | positive 16/18 | unsatisfactory |

NILM means negative for intraepithelial lesion or malignancy

NILM no TZ means negative for intraepithelial lesion or malignancy, but no transformation zone elements present

LSIL means low-grade squamous intraepithelial lesion

ASCUS means atypical squamous cells of undetermined significance

AGC means atypical glandular cells

ASC-H means atypical squamous cells cannot rule out high-grade disease

HSIL means high-grade squamous intraepithelial lesion
