# Supplementary material for: Black US women share their experiences with follow-up after abnormal cervical cancer screening
Source: Public Health Pract (Oxf). 2025 Oct 1;10:100658. doi: 10.1016/j.puhip.2025.100658 (PMC12519130; doi:10.1016/j.puhip.2025.100658)
Supplement: Multimedia component 4 [file mmc4.docx]

Supplemental Table 2. ASCCP 2021-2023 management for 21-29 years

| **Repeat Cytology in one year** | | **Repeat Cytology (reflex HPV testing) in 3 years** | | **Colposcopy with Biopsy and Endocerivcal Curettage** | |
| --- | --- | --- | --- | --- | --- |
| cytology result | HPV result | cytology result | HPV result | cytology result | HPV result |
| Negative for Intraepithelial Lesion or Malignancy (NILM) | positive | Negative for Intraepithelial Lesion or Malignancy (NILM) | unknown/  none | Negative for Intraepithelial Lesion or Malignancy (NILM) but no T-zone | positive 16/18 |
| Negative for Intraepithelial Lesion or Malignancy (NILM) | positive  12 other | Negative for Intraepithelial Lesion or Malignancy (NILM) | negative | Negative for Intraepithelial Lesion or Malignancy (NILM) | positive 16/18 |
| Negative for Intraepithelial Lesion or Malignancy (NILM)  but no T-zone | positive | Negative for Intraepithelial Lesion or Malignancy (NILM)  but no T-zone | negative |  |  |
| Negative for Intraepithelial Lesion or Malignancy (NILM)  but no T-zone | positive  12 other | Negative for Intraepithelial Lesion or Malignancy (NILM)  but no T-zone | unknown/  none |  |  |
| Atypical Squamous Cells of Undetermined Significance (ASCUS) | unknown/  none | Atypical Squamous Cells of Undetermined Significance (ASCUS) | negative | Atypical Squamous Cells of Undetermined Significance (ASCUS) | positive |
|  |  |  |  | Atypical Squamous Cells of Undetermined Significance (ASCUS) | positive 16/18 |
|  |  |  |  | Atypical Squamous Cells of Undetermined Significance (ASCUS) | positive  12 other |
|  |  |  |  | Atypical Squamous Cells -cannot rule out High-Grade disease (ASC-H) | unknown/  none |
|  |  |  |  | Atypical Squamous Cells -cannot rule out High-Grade disease (ASC-H) | negative |
|  |  |  |  | Atypical Squamous Cells -cannot rule out High-Grade disease (ASC-H) | positive |
|  |  |  |  | Atypical Glandular Cells (AGC) | unknown/  none |
|  |  |  |  | Atypical Glandular Cells (AGC) | negative |
|  |  |  |  | Atypical Glandular Cells (AGC) | positive |
| Low-Grade Squamous Intraepithelial Lesion (LSIL) | unknown/  none | Low-Grade Squamous Intraepithelial Lesion (LSIL) | negative | Low-Grade Squamous Intraepithelial Lesion (LSIL) | positive |
| Low-Grade Squamous Intraepithelial Lesion (LSIL) | positive |  |  | Low-Grade Squamous Intraepithelial Lesion (LSIL) | positive 16/18 |
|  |  |  |  | Low-Grade Squamous Intraepithelial Lesion (LSIL) | positive  12 other |
|  |  |  |  | High Grade Squamous Intraepithelial Lesions (HSIL) | unknown/  none |
|  |  |  |  | High Grade Squamous Intraepithelial Lesions (HSIL) | negative |
|  |  |  |  | High Grade Squamous Intraepithelial Lesions (HSIL) | positive |
|  |  |  |  | unsatisfactory | positive 16/18 |
