# Supplementary material for: Black US women share their experiences with follow-up after abnormal cervical cancer screening
Source: Public Health Pract (Oxf). 2025 Oct 1;10:100658. doi: 10.1016/j.puhip.2025.100658 (PMC12519130; doi:10.1016/j.puhip.2025.100658)
Supplement: Multimedia component 5 [file mmc5.docx]

Supplemental Table 3. Demographics of the random population and interviewed population

|  | No Follow-up | | Appropriate Follow-up | | Missed colposcopy but did come for further surveillance | |
| --- | --- | --- | --- | --- | --- | --- |
|  | Random | Interviewed | Random | Interviewed | Random | Interviewed |
|  | N=28 | N=5 | N=20 | N=7 | N=10 | N=2 |
| **Age, yrs,** mean (SD) | 42.2 (11.7) | 46.2 (17.6) | 41.4 (8.6) | 40.3 (10.4) | 43.3 (9.7) | 42.3 (5.8) |
| 21-29 (n=9) | 27.5 (0.8) | 27.1 (0) | 29.6 (0) | 24.7 (0) | 0 (0) | 0 (0) |
| 30-65 (n=63) | 46.2 (9.8) | 51.0 (16.1) | 41.4 (8.6) | 42.9 (8.6) | 43.3 (9.7) | 42.3 (5.8) |
| **Gyn Status** |  |  |  |  |  |  |
| Reproductive | 22 (78.6) | 3 (60.0) | 17 (85.0) | 6 (85.7) | 9 (90.0) | 2 (100) |
| Perimenopause | 1 (3.6) | 0 (0) | 1 (5.0) | 0 (0) | 0 (0) | 0 (0) |
| Menopausal | 5 (12.1) | 2 (40.0) | 2 (10.0) | 1 (14.3) | 1 (10.0) | 0 (0) |
| **Number of medicines** |  |  |  |  |  |  |
| **0** (n=11) | 3 (10.7) | 0 (0) | 4 (20.0) | 2 (28.6) | 0 (0) | 0 (0) |
| **1-4** (n=38) | 15 (53.6) | 3 (60.0) | 8 (40.0) | 2 (28.6) | 6 (60.0) | 0 (0) |
| **5+** (n=37) | 10 (35.7) | 2 (40.0) | 8 (40.0) | 3 (42.9) | 4 (40.0) | 2 (100) |

There were no differences between the random and interviewed populations
